# Locus coeruleus integrity correlates with inhibitory functions of the fronto-subthalamic ‘hyperdirect’ pathway in Parkinson’s disease

**DOI:** 10.1016/j.nicl.2022.103276

**Published:** 2022-11-28

**Authors:** Biman Xu, Tingting He, Yuan Lu, Jia Jia, Barbara J. Sahakian, Trevor W. Robbins, Lirong Jin, Zheng Ye

**Affiliations:** a Institute of Neuroscience, Center for Excellence in Brain Science and Intelligence Technology, Chinese Academy of Sciences, Yueyang Road 320, Shanghai 200031, China; b University of Chinese Academy of Sciences, Yuquan Road 19(A), Beijing 100049, China; c Department of Neurology, Zhongshan Hospital, Fudan University, Fenglin Road 180, Shanghai 200032, China; d Department of Psychiatry, University of Cambridge, Herchel Smith Building for Brain & Mind Sciences, Forvie Site, Robinson Way, Cambridge CB2 0SZ, UK; e Institute of Science and Technology for Brain-Inspired Intelligence, Fudan University, Handan Road 220, Shanghai 200433, China; f Department of Psychology, University of Cambridge, Downing Street, Cambridge CB2 3EB, UK

**Keywords:** Locus coeruleus, Fronto-subthalamic hyperdirect pathway, Response inhibition, Parkinson's disease, Neuromelanin-sensitive MRI, fMRI, CNR, contrast-to-noise ratio, IFG, inferior frontal gyrus, LC, locus coeruleus, PD, Parkinson’s disease, preSMA, pre-supplementary motor area, SN, substantia nigra, SSRT, stop-signal reaction time, STN, subthalamic nucleus, UPDRS, Unified Parkinson’s Disease Rating Scale

## Abstract

A long-running debate concerns whether dopamine or noradrenaline deficiency drives response disinhibition in Parkinson’s disease (PD). This study aimed to investigate whether damage to the locus coeruleus (LC) or substantia nigra (SN) might impact inhibitory functions of the fronto-subthalamic hyperdirect or fronto-striatal indirect pathway. Patients with PD (*n* = 29, 13 women) and matched healthy controls (*n* = 29, 15 women) participated in this cross-sectional study. LC and SN integrity was assessed using neuromelanin-sensitive MRI. Response inhibition was measured using fMRI with a stop-signal task. In healthy controls, LC (but not SN) integrity correlated with the stopping-related activity of the right inferior frontal gyrus (IFG) and right subthalamic nucleus (STN), which further correlated with stop-signal reaction time (SSRT). PD patients showed reduced LC integrity, longer SSRT, and lower stopping-related activity over the right IFG, pre-supplementary motor area, and right caudate nucleus than healthy controls. In PD patients, the relationship between SSRT and the fronto-subthalamic pathway was preserved. However, LC integrity no longer correlated with the stopping-related right IFG or right STN activity. No contribution of SN integrity was found during stopping. In conclusion, LC (but not SN) might modulate inhibitory functions of the right IFG-STN pathway. Damage to the LC might impact the right IFG-STN pathway during stopping, leading to response disinhibition in PD.

## Highlights

• Locus coeruleus integrity correlates with subthalamic activity during stopping.
• Locus coeruleus integrity correlates with frontal activity during stopping.
• Stopping-related frontal and subthalamic activity correlates with stopping speeds.

## Graphical abstract

**Figure:**
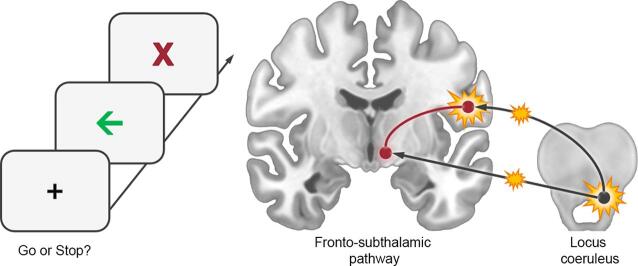

## 1 Introduction

**Fig. 1:**
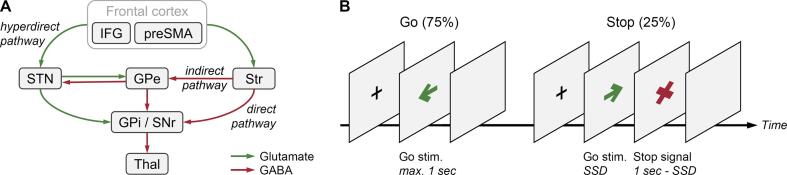
Fronto-basal ganglia loops and the stop-signal task. (**A**) Fronto-basal ganglia loops. IFG, inferior frontal gyrus; preSMA, pre-supplementary motor area; STN, subthalamic nucleus; Str, striatum; GPi, globus pallidus internus; GPe, globus pallidus externus; SNr, substantia nigra pars reticulata; Thal, thalamus. (**B**) The stop-signal task. SSD, stop-signal delay.

The ability to suppress prepotent responses is a core executive function that is impaired in Parkinson’s disease (PD) (Di Caprio et al., 2020, Gauggel et al., 2004). Response inhibition is thought to be supported by fronto-basal ganglia loops, including the fronto-subthalamic hyperdirect and fronto-striatal indirect pathways (Fig. 1A) (Jahanshahi et al., 2015, Nambu et al., 2002). It is often measured as the stop-signal reaction time (SSRT) derived from the stop-signal task (Fig. 1B) (Logan and Cowan, 1984, Verbruggen et al., 2019).

A long-held debate concerns whether dopamine or noradrenaline deficiency (or both) drives response disinhibition in PD. A series of human and animal studies using the selective noradrenaline reuptake inhibitor atomoxetine has shown the role of noradrenaline in response inhibition. In rats, atomoxetine can produce a dose-dependent speeding of SSRT (Bari et al., 2009, Bari et al., 2011, Robinson et al., 2008). In healthy adults and patients with PD, oral administration of atomoxetine can speed SSRT (Chamberlain et al., 2006, O'Callaghan et al., 2021), which is associated with enhanced stopping-related activity over the right inferior frontal gyrus (IFG) (Chamberlain et al., 2009, Ye et al., 2015). However, human and animal studies have also shown a role for dopamine in response inhibition. In rats, blockade of D1 or D2 receptors in the dorsomedial striatum can modulate SSRT (Eagle et al., 2011). In healthy adults, higher availability of dorsostriatal D2/3 receptors predicts faster SSRT (Ghahremani et al., 2012). In patients with PD, overnight withdrawal of levodopa can prolong SSRT, which is associated with reduced stopping-related activity and functional connectivity in the fronto-striatal pathway (Manza et al., 2018). Here we revisit the issue from a different angle: investigating whether the structural integrity of locus coeruleus (LC) or substantia nigra (SN) correlates with inhibitory functions of the fronto-subthalamic hyperdirect or fronto-striatal indirect pathway using neuromelanin-sensitive MRI and fMRI.

Alpha-synuclein pathology affects the LC in stage 2 and the SN in stage 3 of PD (Braak et al., 2003, Braak et al., 2006). LC and SN integrity can be measured *in vivo* using neuromelanin-sensitive MRI. Neuromelanin is a by-product of catecholamine synthesis, existing in LC noradrenaline neurons and SN dopamine neurons (Fedorow et al., 2005, Zecca et al., 2001). In PD, neuromelanin signals of the LC and SN are remarkably diminished (Sasaki et al., 2006, Wang et al., 2018), consistent with the loss of noradrenaline and dopamine neurons in these nuclei (Isaias et al., 2016, Ito et al., 2017).

Taking advantage of multimodal neuroimaging, we examined whether and how damage to the LC or SN might impact the fronto-subthalamic or fronto-striatal pathway during stopping. First, we expected to observe reduced LC and SN integrity and prolonged SSRT in patients with PD. Second, we expected to detect the reduced stopping-related activity of the fronto-subthalamic or fronto-striatal pathway in patients with PD. Third, we aimed to explore relationships between LC integrity, fronto-subthalamic pathway, and SSRT.

## 2 Material and methods

The study was approved by the ethics committee of the Chinese Academy of Sciences Institute of Neuroscience following the Declaration of Helsinki. All participants signed written informed consents before participating in this study.

### 2.1 Patients and clinical assessment

We recruited 29 patients with idiopathic PD at the Zhongshan Hospital Department of Neurology between 2020 and 2021. Inclusion criteria were (a) PD diagnosed by senior neurologists according to the Movement Disorder Society clinical diagnostic criteria for PD (Postuma et al., 2015), (b) Hoehn-Yahr 1–3, (c) age 50–75 years, (d) education > 6 years, and (e) right-handed. Exclusion criteria were (a) a history of other neurological or psychiatric diseases, (b) alcohol or drug abuse, (c) possible current depression (Geriatric Depression Scale > 10/30) or intake of antidepressants, (d) possible dementia (Montreal Cognitive Assessment Basic, MoCA-B < 21/30) or intake of antidementia drugs, or (e) contraindications to MRI. All patients were assessed on their regular dopaminergic drugs, including levodopa (*n* = 20), pramipexole (*n* = 16), piribedil (*n* = 8), rasagiline (*n* = 8), selegiline (*n* = 2), and amantadine (*n* = 5).

Table 1 shows the demographic, clinical, and neuropsychological data. Ten additional patients were excluded due to incompletion of the stop-signal task (*n* = 2), violation of the race model for computing SSRT (*n* = 1), excessive head motion (*n* = 6), or feeling of discomfort during scanning (*n* = 1).

**Table 1:** Demographic, clinical, and neuropsychological data (means, SDs, and group differences).

Features/Measures	PD (*n* = 29)	HC (*n* = 29)	Group differences (*p* values)
Male/Female	16/13	14/15	0.599
Age (years)	64.9 (6.7)	64.3 (5.5)	0.684
Education (years)	10.4 (2.4)	11.3 (1.6)	0.100
Body Mass Index	23.5 (3.7)	23.6 (2.9)	0.869
*Motor symptoms*			
Age of onset (years)	63.0 (6.1)	–	–
Disease duration (years)	2.5 (2.3)	–	–
Hoehn and Yahr stage	1.8 (0.7)	–	–
UPDRS III score	25.1 (12.4)	–	–
*Levodopa equivalent daily dose*			
Total (mg/day)	301.8 (184.2)	–	–
Levodopa (mg/day)	177.6 (142.3)	–	–
D2/3 receptor agonists (mg/days)	68.5 (49.5)	–	–
*Non-motor functions*			
Geriatric Depression Scale score	5.3 (2.8)	3.9 (2.7)	0.060
REM Sleep Behavior Disorder Screening Questionnaire score	5.5 (3.5)	1.8 (1.4)	<0.001*
Epworth Sleep Scale score	5.9 (4.9)	4.1 (3.5)	0.112
Montreal Cognitive Assessment Basic score	25.8 (1.9)	28.1 (1.1)	<0.001*

Group differences, *p* values of two-sample *t* or Chi-square tests as appropriate; asterisks, *p* < 0.007 (Bonferroni correction for eight tests); UPDRS, Unified Parkinson’s Disease Rating Scale.

### 2.2 Healthy controls

We recruited 29 healthy controls (HCs). Inclusion criteria were (a) age 50–75 years, (d) education > 6 years, and (c) right-handed. Exclusion criteria were (a) a history of significant neurological or psychiatric diseases, (b) alcohol or drug abuse, (c) possible current depression, (d) possible mild cognitive impairment (MoCA-B < 26/30), or (e) contraindications to MRI. Three additional HCs were excluded due to a violation of the race model for computing SSRT.

### 2.3 Stop-signal task

The stop-signal task was designed, analyzed, and reported following a recent consensus (Verbruggen et al., 2019). The task included interleaved 192 Go trials and 64 Stop trials (Fig. 1B). In Go trials, participants responded to the direction of green arrows by pressing left/right buttons with their right hands. In Stop trials, the arrow was replaced by a red “X” after a variable stop-signal delay, instructing participants to cancel the response. The stop-signal delay was adjusted continuously in steps of 50 ms via a standard adaptive tracking procedure to maintain the probability of responding [p(respond|stop-signal)] close to 0.50. The intertrial interval was varied between 2.5 and 5 sec. The trials were pseudo-randomized to ensure that (a) there was no consecutive Stop, and (b) there were no more than three repetitions of left/right responses in consecutive trials.

### 2.4 Analysis of stop-signal task performance

We controlled behavioral data quality by monitoring whether the task performance met the independence assumption of the race model: (a) p(respond|stop-signal) is between 0.25 and 0.75, and (b) the reaction time of failed Stop is numerically shorter than the reaction time of Go (Verbruggen et al., 2019). All included participants met the race-model assumption.

We estimated SSRT using a modified integration method that replaces Go omissions with the maximum reaction time, given the presence of Go omissions (Verbruggen et al., 2019). We reported all relevant parameters, including the rate of Go omissions, rate of Go commission errors, reaction time of correct Go, p(respond|stop-signal), mean stop-signal delay, reaction time of failed Stop, and SSRT (Table 2). Statistical analysis was performed with IBM SPSS Statistics 21. We examined group differences in task performance using one-tailed two-sample *t* tests (PD > HC, *p <* 0.008 Bonferroni correction for seven tests).

**Table 2:** Stop-signal task performance (means, SEMs, and group differences).

Parameters	PD (*n* = 29)	HC (*n* = 29)	Group differences (*p* values)
Rate of Go omissions (%)	3.6 (1.2)	0.9 (0.2)	0.016
Rate of Go commission errors (%)	3.4 (0.8)	1.5 (0.5)	0.023
Reaction time of correct Go (ms)	594 (17)	582 (12)	0.289
P(respond | stop-signal)	0.47 (0.01)	0.46 (0.01)	0.224
Stop-signal delay (ms)	269 (16)	287 (12)	0.181
Reaction time of failed Stop (ms)	538 (16)	518 (11)	0.148
Stop-signal reaction time (ms)	316 (15)	274 (7)	0.007*

Group differences, *p* values of one-tailed two-sample *t* tests (PD > HC); asterisks, *p* < 0.008 (Bonferroni correction for seven tests).

### 2.5 Acquisition of neuroimaging data

Neuroimaging data were acquired on a Siemens Tim Trio 3T MRI scanner with a 32-channel head coil at the Brain Imaging Center of the Center for Excellence in Brain Science and Intelligence Technology.

Structural T1-weighted images used a magnetisation-prepared rapid gradient-echo sequence (192 sequential sagittal slices, repetition time 2300 ms, echo time 3 ms, flip angle 9°, field of view 256 × 256 mm^2^, resolution 1 × 1 × 1 mm^3^).

Neuromelanin-sensitive T1-weighted images used a fast spin-echo sequence with magnetization transfer pulse (16 interleaved axial slices, repetition time 1000 ms, echo time 13 ms, flip angle 90°, field of view 256 × 256 mm^2^, resolution 0.6 × 0.5 × 2.5 mm^3^).

Functional T2*-weighted images used a standard echo-planar imaging sequence (47 interleaved ascending axial slices, repetition time 3000 ms, echo time 30 ms, flip angle 90°, field of view 192 × 192 mm^2^, resolution 2 × 2 × 3 mm^2^).

### 2.6 Preprocessing and analysis of fMRI data

fMRI data were processed using SPM12 (v7219, https://www.fil.ion.ucl.ac.uk/spm). The first three images were discarded to allow magnetization equilibration. All other images were corrected for slice acquisition time difference, realigned to a mean functional image, registered to the high-resolution T1-weighted image, normalized to the Montreal Neurological Institute (MNI) coordinate system, smoothed with a Gaussian kernel of 4-mm full-width half-maximum, and filtered with a 128-sec high-pass filter.

We controlled fMRI data quality by monitoring the total displacement (Wilke, 2012) and spatial normalization (visual inspection). The two groups were similar in the total displacement (two-sample *t* test, *p =* 0.136).

First, we computed the stopping-related regional activation. The general linear model convolved a design matrix with a canonical hemodynamic response function at the subject level. The design matrix included successful Stop, failed Stop, correct Go, Go commission errors, and Go omissions as separate regressors. Each trial was time-locked to its onset. The total displacement was included as a nuisance regressor. Classical parameter estimation was applied with a one-lag autoregressive model. The stopping effect was defined as successful Stop *versus* correct Go (SS > Go). A whole-brain two-sample *t* test was conducted at the group level (voxel-level *p <* 0.001, cluster-level *p <* 0.05 familywise error correction).

Second, we examined whether PD patients showed lower stopping-related activity than HCs in the fronto-subthalamic or fronto-striatal pathway. Regions of interest were derived from a meta-analysis of 99 stop-signal task fMRI studies (NeuroSynth) (Yarkoni et al., 2011), including the right IFG, pre-supplementary motor area (preSMA), right subthalamic nucleus (STN), and right caudate nucleus. The STN and caudate regions were validated using a human basal ganglia atlas (Prodoehl et al., 2008). Stopping-related percent signal change (SS > Go) was computed and entered into one-tailed two-sample *t* tests (PD < HC, *p <* 0.05).

Third, we explored relationships between LC integrity, fronto-subthalamic pathway, and SSRT. We examined: (a) whether SSRT correlated with the stopping-related activity of the fronto-subthalamic or fronto-striatal pathway (stepwise regression, *p <* 0.05), and (b) whether the stopping-related activity of the fronto-subthalamic pathway correlated with LC or SN integrity or levodopa equivalent daily dose (stepwise regression, *p <* 0.05).

### 2.7 Analysis of neuromelanin signals

Two independent, trained raters blind to fMRI data evaluated neuromelanin data quality, excluding the data with excessive head motion or other artifacts (B.X. and Y.L.). They manually identified the LC and SN in the native space to avoid potential distortion in normalization following previous studies (Li et al., 2019, Liu et al., 2021, Prasuhn et al., 2021).

The bilateral LC (circular regions of interest, 2 mm^2^ each) and pontine (20 mm^2^) were identified adjacent to the fourth ventricle in three contiguous slices. The LC contrast-to-noise ratio (CNR) was the difference between the mean LC signal intensity and mean pontine signal intensity divided by the SD of the pontine signal intensity (Li et al., 2019). The LC CNR was averaged across sides.

The SN was identified through a semi-automatical procedure to minimize subjective bias (Liu et al., 2021). The SN was a cluster of voxels with a signal intensity that was 2.5 SDs above the mean signal intensity of the ipsilateral cerebral peduncle region (10 mm^2^ each). The SN area was summed up across sides.

Measurements of the two raters were highly consistent (intraclass correlation coefficient: LC, *r* = 0.98, *p* < 0.001; SN, *r* = 0.98, *p <* 0.001). Their mean measurements were used for statistical analysis. We examined whether PD patients showed reduced LC or SN integrity than HCs using one-tailed two-sample *t* tests (PD < HC, *p <* 0.025 Bonferroni correction for two tests).

## 3 Results

### 3.1 Group differences in LC and SN integrity

**Fig. 2:**
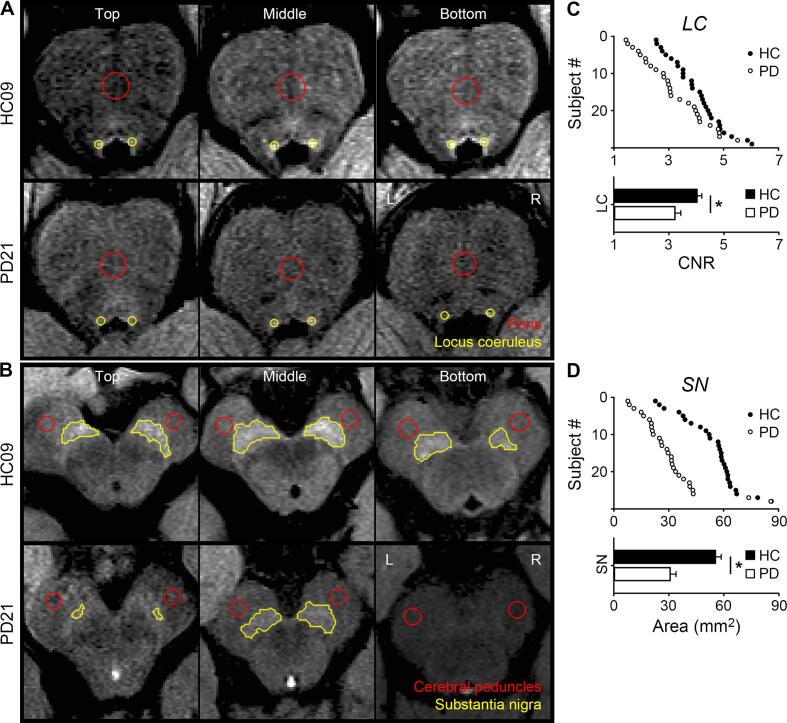
Locus coeruleus (LC) and substantia nigra (SN) integrity. (**A**) LC and pontine and (**B**) SN and cerebral peduncle in three contiguous slices of representative subjects. L, left; R, right. Individual data, group means, and SEMs of (**C**) the LC contrast-to-noise ratio (CNR) and (**D**) SN area in healthy controls (HC) and patients with Parkinson’s disease (PD). Asterisks, *p* < 0.025.

Fig.2A-B shows LC and SN in representative subjects. Fig. 2C-D shows LC and SN integrity. PD patients showed reduced LC (one-tailed two-sample *t* tests, *t*(55) = −2.92, *p* = 0.002) and SN integrity than HCs (*t*(55) = −5.33, *p* < 0.001). The group difference in LC integrity was confirmed by an atlas-based analysis (see supplement) (Ye et al., 2021a).

### 3.2 Group differences in SSRT

Table 2 shows the stop-signal task performance. PD patients showed longer SSRT, but not longer Go reaction times, than HCs. Across groups, SSRT did not correlate with the Montreal Cognitive Assessment Basic score (*p* = 0.42) or Geriatric Depression Scale score (*p* = 0.97).

### 3.3 Group differences in stopping-related activity

**Fig. 3:**
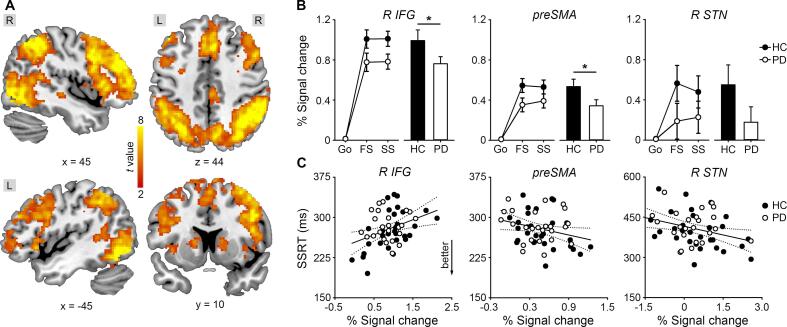
Stopping-related activity. (**A**) Stopping-related regional activation across groups (voxel-level *p* < 0.001, cluster-level *p* < 0.05 familywise error correction). Color scale indicates *t* values. Coordinates are in the MNI space. L, left; R, right. (**B**) Scatter plots show group means and SEMs of the percent signal change for correct Go, successful Stop (SS), and failed Stop (FS) in healthy controls (HC) and patients with Parkinson’s disease (PD). Bar plots show group means and SEMs of the stopping-related activity (SS > Go). IFG, inferior frontal gyrus; preSMA, pre-supplementary motor area; STN, subthalamic nucleus; asterisks, *p <* 0.05. (**C**) SSRT correlated with the stopping-related activity across groups. Solid lines, *p <* 0.05; dotted lines, 95% confidence intervals.

Fig. 3A and Table 3 show the stopping-related regional activation across groups (SS > Go, whole-brain two-sample *t* test). The IFG, preSMA, and caudate nucleus were more activated for successful Stop than correct Go. The STN did not show significant stopping-related regional activation at the group level, probably due to a larger inter-individual variability. Given the importance of the fronto-subthalamic pathway, we applied region-of-interest analysis to the STN.

**Table 3:** Stopping-related regional activation across groups (SS > Go).

Regions	Brodmann areas	Sides	Peaks in MNI (x, y, z)	*t* values	Cluster sizes (voxels)
Pre-supplementary motor area /supplementary motor area	6/8/32	L/R	[4, 22, 46]	9.12	1204
Inferior frontal gyrus	44/45	L	[–32, 28, 2]	8.45	1265
		R	[50, 16, 34]	9.95	2475
Insula	–	L	[-30, 20, 6]	10.02	528
		R	[36, 18, 4]	11.53	447
Inferior parietal lobule	7/40	L	[-34, −62, 50]	10.03	1419
		R	[38, −52, 44]	10.64	855
Middle cingulate gyrus	23/24	L/R	[0, −28, 30]	7.56	377
Middle occipital gyrus	18	L	[-18, −96, 4]	7.84	1794
		R	[28, −90, 14]	11.09	1166
Caudate nucleus	–	L	[-12, 4, 8]	6.49	149
		R	[14, 10, 10]	5.13	87
Thalamus	–	R	[12, −12, 12]	6.20	136

MNI, Montreal Neurological Institute coordinate system; L, left; R, right.

PD patients showed lower stopping-related activity than HCs in the fronto-subthalamic and fronto-striatal pathways (Fig. 3B), including the right IFG (one-tailed two-sample *t* tests, *t*(56) = −1.81, *p =* 0.038), preSMA (*t*(56) = −1.99, *p =* 0.025), and right caudate nucleus (*t*(56) = −2.25, *p =* 0.015), and marginally in the right STN (*t*(56) = −1.50, *p =* 0.07). However, these tests were only significant using one-tailed *t* tests and not corrected for multiple comparisons.

### 3.4 Brain-behavior relationships

First, SSRT correlated with the stopping-related activity of the right IFG, preSMA, and right STN across groups (Fig. 3C). A stepwise regression model for SSRT (*F*(3,50) = 5.04, *p =* 0.004, *R*^2^ = 0.23) included the stopping-related right IFG (*t* = 2.80, *p =* 0.007), preSMA (*t* = −2.02, *p =* 0.048) and right STN activity (*t* = −2.46, *p =* 0.017) but excluded the stopping-related caudate activity (*p* = 0.78). Four outliers were analyzed separately (see supplement). Participants with higher stopping-related preSMA and right STN activity and lower stopping-related right IFG activity showed shorter SSRT.

**Fig. 4:**
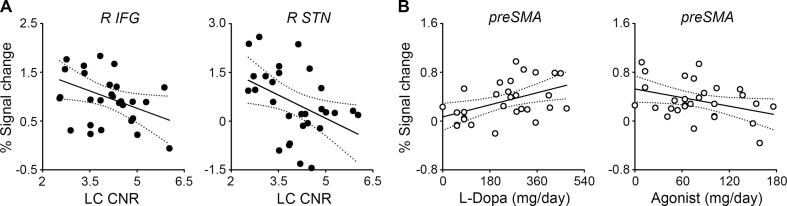
Relationship between locus coeruleus (LC) integrity, dopaminergic drugs, and stopping-related activity. (**A**) LC contrast-to-noise ratio (CNR) correlated with the stopping-related activity of the right inferior frontal gyrus (IFG) and subthalamic nucleus (STN) in healthy controls. Solid lines, *p <* 0.05; dotted lines, 95% confidence intervals. (**B**) Daily levodopa dose and levodopa equivalent daily dose of D2/3 receptor agonists correlated with the stopping-related pre-supplementary motor area activity (preSMA) in patients with Parkinson’s disease.

Second, LC (but not SN) integrity correlated with the stopping-related activity of the right IFG and right STN in HCs (Fig. 4A). A stepwise regression model for the stopping-related right IFG activity (*F*(1,27) = 4.76, *p =* 0.038, *R*^2^ = 0.15) included the LC CNR (*t* = −2.18, *p =* 0.038) but excluded the SN area (*p =* 0.757). A stepwise regression model for the stopping-related right STN activity (*F*(1,27) = 5.81, *p =* 0.023, *R*^2^ = 0.18) also included the LC CNR (*t* = −2.41, *p =* 0.023) but not the SN area (*p =* 0.466). HCs with better LC integrity showed higher stopping-related right IFG and right STN activity. However, no such correlation was found for PD patients (*p*s > 0.27).

Third, daily doses of dopaminergic drugs correlated with the stopping-related preSMA activity in PD patients (Fig. 4B). A stepwise regression model for the stopping-related preSMA activity (*F*(2,25) = 4.33, *p =* 0.024, *R*^2^ = 0.26) included the daily levodopa dose (*t* = 2.49, *p =* 0.020) and levodopa equivalent daily dose of D2/3 receptor agonists (*t* = −2.15, *p =* 0.041) but excluded the LC CNR and SN area (*p*s > 0.38). PD patients with daily exposure to more levodopa and fewer receptor agonists showed higher stopping-related preSMA activity.

## 4 Discussion

**Fig. 5:**
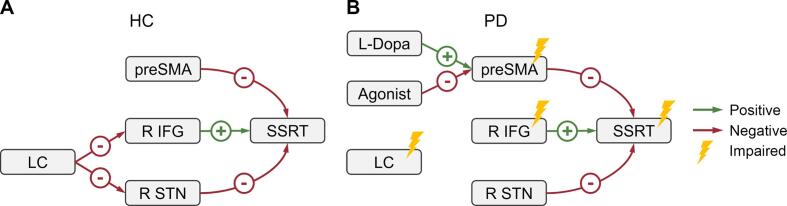
Schematic diagram of the relationship between the locus coeruleus (LC), dopaminergic drugs, fronto-subthalamic pathway, and SSRT. (**A**) Healthy controls (HC). (**B**) Patients with Parkinson’s disease (PD). Green and red arrows indicate positive and negative correlations, respectively. Yellow markers indicate structural or functional impairment. (For interpretation of the references to color in this figure legend, the reader is referred to the web version of this article.)

Cognitive decline in PD correlates with the progression of α-synuclein pathology (Braak et al., 2003, Braak et al., 2006). Causal contributions of LC and SN neurodegeneration from stages 2–3 onwards may be critical for PD's early manifestations of executive dysfunction (Li et al., 2019, Prasuhn et al., 2021). We found that LC (but not SN) structural integrity correlated with inhibitory functions of the right IFG-STN hyperdirect pathway using neuromelanin-sensitive MRI and fMRI with a stop-signal task. In HCs (Fig. 5A), LC integrity correlated with the stopping-related right IFG and right STN activity, which further correlated with SSRT. In contrast, the stopping-related preSMA activity correlated with SSRT but not LC integrity.

Damage to the LC may impact the right IFG-STN hyperdirect pathway during stopping. In PD patients (Fig. 5B), the relationship between SSRT and the fronto-subthalamic pathway was preserved. However, LC integrity was reduced and no longer associated with the stopping-related right IFG or right STN activity. PD patients showed prolonged SSRT and reduced stopping-related right IFG and preSMA activity. No contribution of SN integrity was found. Instead, an impact of levodopa and D2/3 receptor agonists was present. Daily exposure to more levodopa and less D2/3 receptor agonists correlated with greater stopping-related preSMA activity. No effect of tissue volume loss or lateralization of motor symptoms was found (see supplement).

### 4.1 Stopping and fronto-subthalamic hyperdirect pathway

The STN is a strong candidate for inhibiting an initiated response within hundreds of milliseconds as the conduction velocity of ipsilateral cortico-subthalamic axons is ∼ 7 m/s, much faster than that of the cortico-striatal axons (∼1.5 m/s) (Mathai and Smith, 2011). The STN is thought to send a global NoGo signal to suppress all responses rather than a particular response when multiple competing responses are activated (Frank, 2006). Consistent with this hypothesis, we found that greater stopping-related right STN activity correlated with faster SSRT (Aron et al., 2007, Aron and Poldrack, 2006).

Beyond the STN, we observed a complex regulation of response inhibition in frontal regions projecting along the hyperdirect pathway. In healthy adults and PD patients, higher stopping-related preSMA activity and lower stopping-related right IFG activity correlated with faster SSRT. This observation is in line with diffusion MRI findings that both right IFG-STN and preSMA-STN white matter tracts are engaged in response inhibition (Coxon et al., 2016, Rae et al., 2015). However, the observed relationship between SSRT and stopping-related right IFG activity is inconsistent with a previous finding in healthy young adults (Aron et al., 2007). It suggests that greater right IFG activity might reflect less efficient inhibition in older adults and PD patients.

It remains unclear which fronto-subthalamic pathway is dominant or how the two fronto-subthalamic pathways interact during stopping. Recent human studies with invasive EEG and single-unit recordings have emphasized the importance of the right IFG-STN pathway (Chen et al., 2020, Mosher et al., 2021). Chen et al. confirmed the existence of a monosynaptic projection from the IFG to ventral STN, which was found by earlier anterograde tracer studies in non-human primates (Haynes and Haber, 2013). Better synchronization between the IFG and ventral STN task-evoked potentials predicts faster SSRT (Chen et al., 2020). Moreover, Mosher et al. showed that ventral STN neurons are activated at short latencies close to SSRT after the stop-signal onset (Mosher et al., 2021). The findings suggest that the monosynaptic pathway from the IFG to ventral STN is critical for fast stopping.

In contrast, human imaging studies suggested that the right IFG modulates the excitatory projection from the preSMA to STN (Jahanshahi et al., 2015). Rae et al. found that stronger preSMA-STN connectivity and greater excitatory IFG modulation correlated with faster SSRT (Rae et al., 2015). However, we obtained no significant correlation between the right IFG activity and prSMA-STN functional connectivity (see supplement).

### 4.2 Noradrenergic modulation of the IFG-STN hyperdirect pathway

Our novel finding is that greater LC integrity correlated with greater stopping-related right IFG and right STN activity in HCs. This finding thus fills an important gap in the literature. Human imaging studies have shown that the elevation of extracellular noradrenaline levels (via intake of selective noradrenaline reuptake inhibitors or genetic variation of a noradrenaline transporter gene) can enhance stopping-related right IFG activity (Chamberlain et al., 2009, Whelan et al., 2012, Ye et al., 2015). Population-based, cohort studies have reported a relationship between LC integrity and noradrenergic-dependent functions (including response inhibition) (Liu et al., 2020, Tomassini et al., 2022). Therefore, we propose that the LC-noradrenergic system modulates the right IFG-STN pathway during stopping.

### 4.3 Dopaminergic modulation of the preSMA

SN integrity did not contribute to the preSMA-STN pathway (also see supplement). However, levodopa and D2/3 receptor agonists may impact the stopping-related preSMA activity in PD patients. PD patients with daily exposure to more levodopa and fewer D2/3 receptor agonists showed higher stopping-related preSMA activity, potentially leading to faster SSRT. This observation is consistent with previous findings that both D1 and D2 receptors can modulate SSRT in humans and animals (Eagle et al., 2011, Manza et al., 2018), and that D2/3 receptor agonist therapy may lead to behavioral disinhibition and impulsivity in PD patients (Dodd et al., 2005).

## 5 Limitations

First, LC and STN are small structures, but the spatial resolution of 3T MRI was limited. Further research with ultra-high field MRI or brain stimulation is needed to confirm our findings and determine the contribution of different LC and STN subregions (Forstmann et al., 2012, Ye et al., 2021a, Ye et al., 2021b). Second, Fig. 5 summarizes the relationship between the LC, fronto-subthalamic hyperdirect pathway, and SSRT. This model was based on correlational evidence from a small sample (*n* = 58). Causal evidence is needed to confirm the model or to investigate hidden factors driving the correlations.

## 6 Conclusions

This study demonstrated that damage to the LC (but not SN) might impact the fronto-subthalamic hyperdirect pathway during stopping. In HCs, LC integrity correlated with the stopping-related right IFG and right STN activity, which further correlated with SSRT. PD patients showed reduced LC integrity, longer SSRT, and lower stopping-related activity over the right IFG and preSMA than HCs. In PD patients, the relationship between SSRT and the fronto-subthalamic pathway was preserved. However, LC integrity no longer correlated with the stopping-related right IFG or right STN activity. No contribution of SN integrity was found during stopping. LC might modulate inhibitory functions of the right IFG-STN pathway. Damage to the LC might impact the right IFG-STN pathway during stopping, leading to response disinhibition in PD.

## CRediT authorship contribution statement

**Biman Xu:** Methodology, Investigation, Formal analysis, Writing – original draft. **Tingting He:** Investigation, Writing – review & editing. **Yuan Lu:** Investigation, Formal analysis, Writing – review & editing. **Jia Jia:** Investigation, Writing – review & editing. **Barbara J. Sahakian:** Writing – review & editing. **Trevor W. Robbins:** Writing – review & editing. **Lirong Jin:** Conceptualization, Resources, Writing – review & editing, Funding acquisition. **Zheng Ye:** Conceptualization, Methodology, Formal analysis, Writing – original draft, Funding acquisition.

## Declaration of Competing Interest

The authors declare that they have no known competing financial interests or personal relationships that could have appeared to influence the work reported in this paper.

## Data Availability

Data will be made available on request.
