# Supplementary material for: Locus coeruleus integrity correlates with inhibitory functions of the fronto-subthalamic ‘hyperdirect’ pathway in Parkinson’s disease
Source: Neuroimage Clin. 2022 Nov 28;36:103276. doi: 10.1016/j.nicl.2022.103276 (PMC9723406; doi:10.1016/j.nicl.2022.103276)
Supplement: Supplementary data 1 [file mmc1.docx]

Supplementary Information for

**Locus coeruleus integrity correlates with inhibitory functions of the fronto-subthalamic ‘hyperdirect’ pathway in Parkinson’s disease**

Biman Xu, BSc,^a,b,†^ Tingting He, MD,^c,†^ Yuan Lu,^a^ Jia Jia, MD,^c^ Barbara J. Sahakian, PhD,^d,e^ Trevor W. Robbins, PhD,^f^,^e^ Lirong Jin, MD, PhD,^c,*^ Zheng Ye, PhD,^a,*^

^a^*Institute of Neuroscience, Center for Excellence in Brain Science and Intelligence Technology, Chinese Academy of Sciences, Yueyang Road 320, Shanghai 200031, China*

^b^*University of Chinese Academy of Sciences, Yuquan Road 19(A), Beijing 100049, China*

^c^*Department of Neurology, Zhongshan Hospital, Fudan University,* *Fenglin Road 180, Shanghai 200032, China*

^d^*Department of Psychiatry, University of Cambridge, Herchel Smith Building for Brain & Mind Sciences, Forvie Site, Robinson Way, Cambridge CB2 0SZ, UK*

*^e^Institute of Science and Technology for Brain-Inspired Intelligence, Fudan University, Handan Road 220, Shanghai 200433, China*

^f^*Department of Psychology, University of Cambridge, Downing Street, Cambridge CB2 3EB, UK*

^†^These authors contributed equally to this work.

*Correspondence to Z. Ye ([yez@ion.ac.cn](mailto:yez@ion.ac.cn)) or L. Jin (jinlr99@163.com)

1. **Atlas-based analysis of LC integrity**

We also analyzed the LC integrity with a probabilistic LC atlas from 7T MRI (Ye et al., 2021). First, for each subject, neuromelanin images were coregistered to skull-stripped T1 images (FreeSurfer v7.1.0, SFig.1A), normalized to the MNI coordinate system, and resampled to voxels of 0.5 × 0.5 × 0.5 mm^3^. LC neuromelanin signals were extracted from each slice using the probabilistic atlas with 5% (88.1 mm^3^) and 25% thresholds (35.5 mm^3^) (SFig.1B). Second, normalized neuromelanin images were group-averaged to identify the pontine (reference area, SFig.1C). A cubic region of interest was placed at the central pontine area of the group-averaged neuromelanin image (4 × 4 × 4.5 mm^3^). Third, for each subject, the LC CNR was computed slice by slice as the difference between the mean LC signal intensity and mean pontine signal intensity divided by the SD of the pontine signal intensity (Li et al., 2019). The LC CNR was averaged across sides.

The group difference in LC integrity was confirmed with the 5% threshold (one-tailed two-sample *t* tests, *t*(55)=-2.08, *p*=0.021) but not with the 25% threshold (*p*=0.096) (SFig.2A). The lack of significance with the 25% threshold was likely due to a large inter-individual variability (SFig.2B).

Finally, we examined the consistency between the atlas-based (MNI space) and manual analysis (native space). Both methods revealed the group difference in LC integrity. LC CNR values from the two methods were highly correlated (Pearson correlation, 5% threshold, HC: *r*=0.65, *p*<0.001, PD: *r*=0.80, *p*<0.001) (SFig.3). However, we did not obtain correlations between the atlas-based LC CNR and stopping-related brain activity (*p*s>0.12). One possibility is that in the atlas-based analysis, neuromelanin signals were distorted in normalization, leading to non-significance.

**SFig.1:** (A) Coregistration and normalization of individual neuromelanin (NM) images. (B) Application of the probabilistic atlas to individual NM images. Prob5, 5% threshold; Prob25, 25% threshold. (C) Group-averaged normalized NM images and identification of the pontine (red).


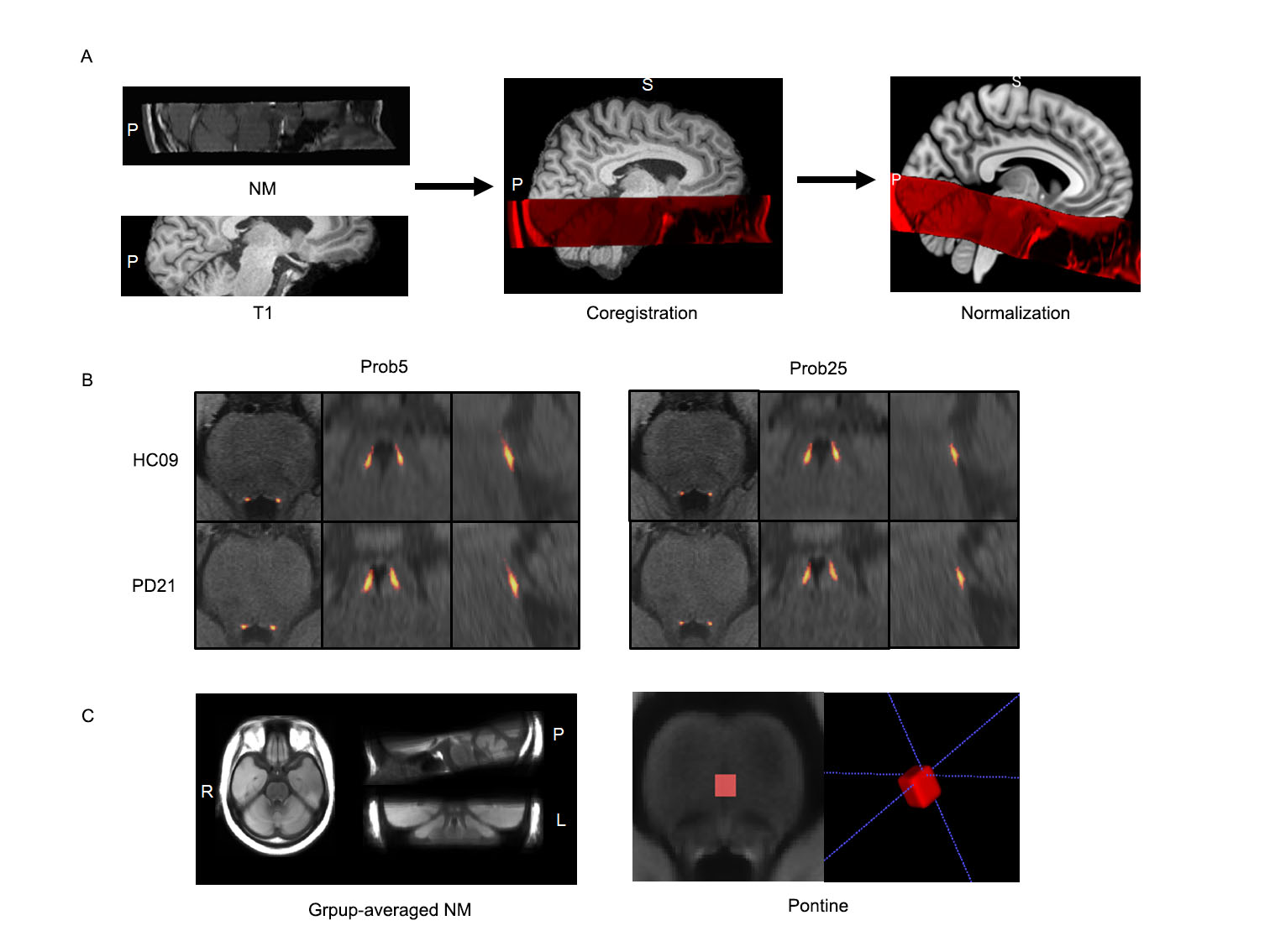


**SFig.2:** (A) Group means and SEMs of the LC contrast-to-noise ratio (CNR) from the probabilistic atlas with 5% (Prob5) and 25% thresholds (Prob25) in healthy controls (HC) and patients with Parkinson’s disease (PD). (B) Slice-by-slice LC CNR along the caudal-rostral axis. The dotted lines indicate standard deviations.


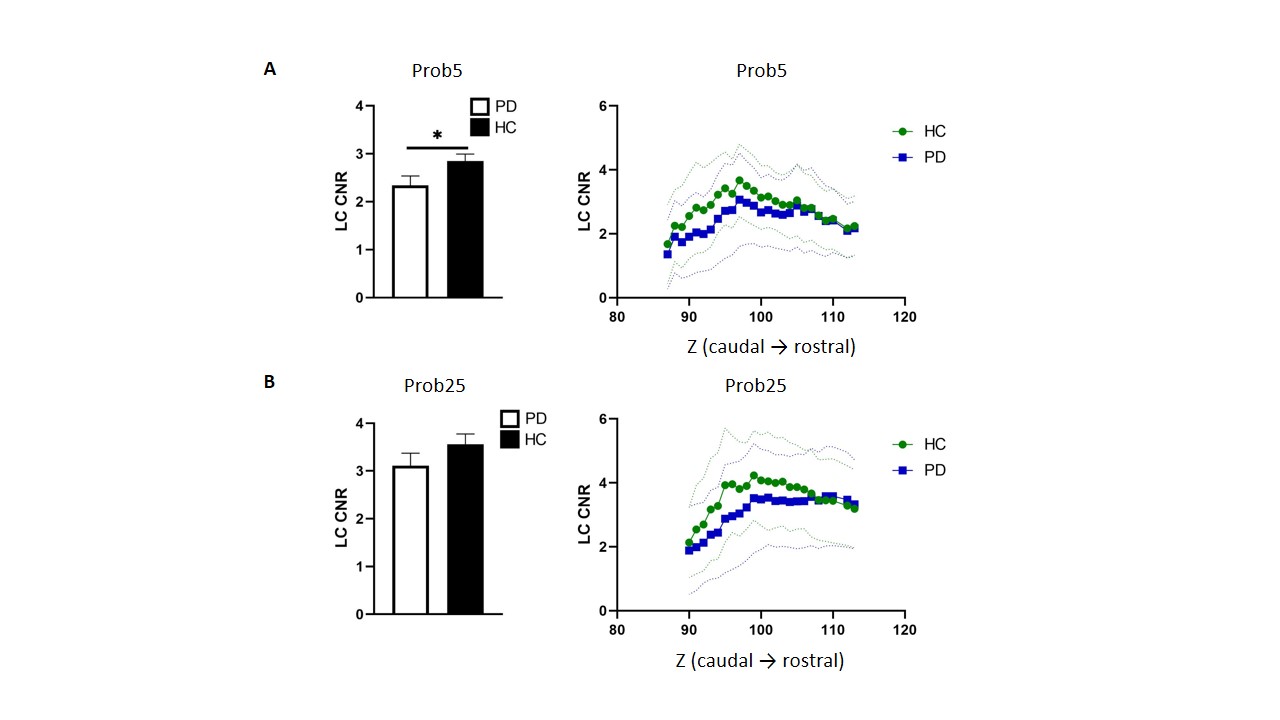


**SFig.3:** High correlations between the LC CNR values from the atlas-based analysis and manual analysis. (A) 5% threshold. (B) 25% threshold.


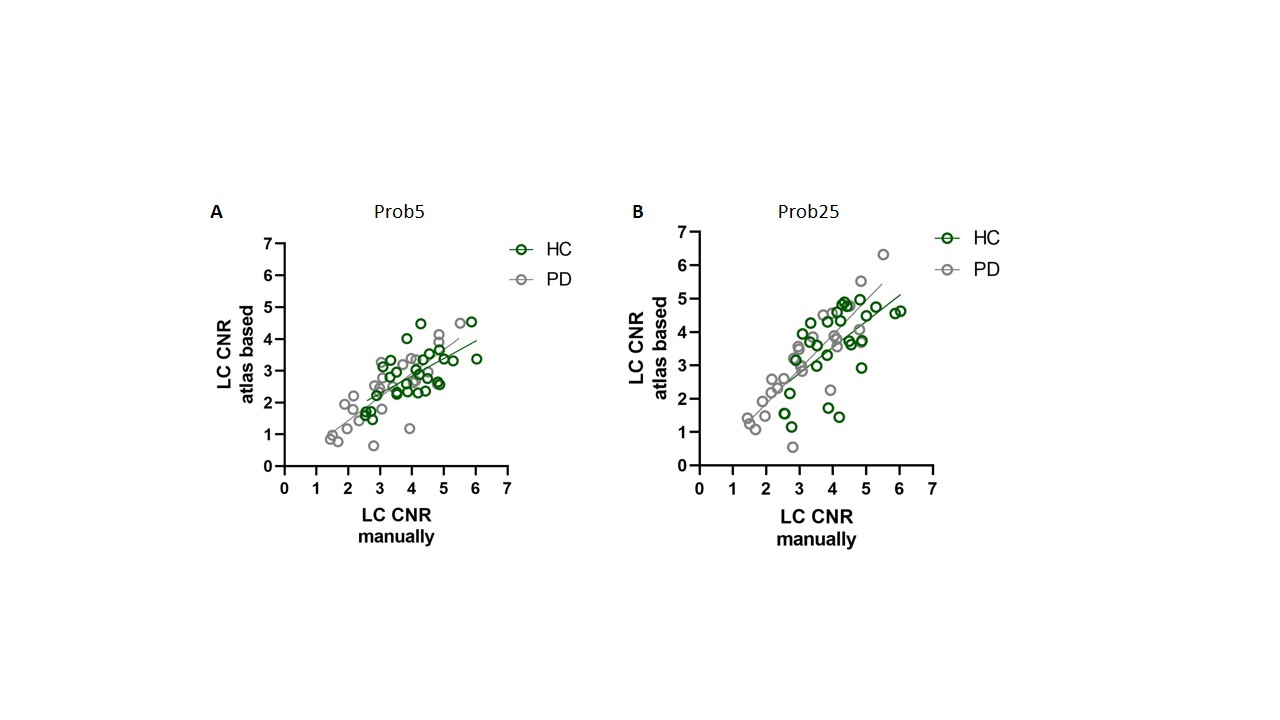


1. **Analysis of PD outliers**

Four PD outliers did not fit the linear regression model across groups (SFig.4A). We then performed a separate linear regression model in PD patients to explore the brain-behavior relationship. However, the outliers led to misleading results (SFig.4B).

For PD patients, the stepwise regression model for SSRT (*F*(1,27)=4.44, *p*=0.045, *R*^2^=0.14) included functional connectivity between the preSMA and right STN (*t*=-2.11, *p*=0.045) but not other factors. The stopping-related activity of the right IFG (*p*=0.156), preSMA (*p*=0.068), and right STN (*p*=0.948) were marginally or not significant. However, the effect of the preSMA-right STN functional connectivity was driven by the outliers. The effect was absent when the outliers were excluded.

**SFig.4:** (A) Four PD outliers (blue dots). (B) Misleading correlation between SSRT and preSMA-right STN functional connectivity.


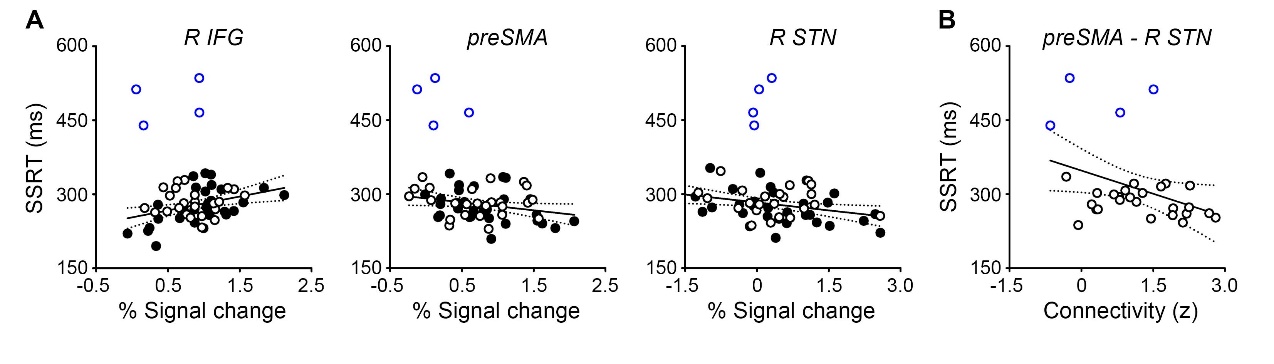


1. **No association between stopping-related activity and tissue volume**

We examined the effect of tissue volume on stopping-related activity in the fronto-subthalamic and fronto-striatal pathways. In particular, we asked (a) whether regions with group differences in the stopping-related activity also showed group differences in the tissue volume; and (b) whether the stopping-related activity correlated with the tissue volume.

High-resolution T1-weighted images were processed with an open-source software FreeSurfer (version 7.1) using a standard cortical reconstruction pipeline.(Fischl, 2012) T1 images were corrected for head motion, normalized to reduce signal intensity non-uniformity and fluctuation, and transformed into the Talairach space. Non-brain tissues (e.g., skull and neck) were removed. White matter and deep grey matter nuclei were segmented using an automated algorithm. Regions of interest were parcellated according to the Destrieunx atlas,(Destrieux, Fischl, Dale, & Halgren, 2010) including the preSMA (superior frontal gyrus, anterior cingulate gyrus and sulcus, and anterior part of the middle cingulate gyrus and sulcus), right IFG (pars opercularis and pars triangularis), and right caudate nucleus. Note, the STN is not labeled in the atlas. The tissue volume of each region was computed for further analysis.

First, we examined group differences in the tissue volume using two-sample *t* tests (*p*<0.017 Bonferroni correction for three tests). Consistent with a recent meta-analysis of 41 MRI studies,(Minkova et al., 2017) PD patients did not show a consistent volumetric reduction (STable 1).

Second, we found no significant correlation between the tissue volume and stopping-related activity in the preSMA (*p*=0.367) or right IFG (*p*=0.107). The correlation was not significant in the right caudate nucleus after removing an outlier (*p*=0.505, SFig.5).

Therefore, we found no effect of tissue volume on stopping-related activity in either fronto-subthalamic or fronto-striatal pathways.

**STable 1:** Tissue volumes (mm^3^) (means, SDs, and group differences)

| Regions | Sides | PD  (*n*=29) | HC  (*n*=29) | Group differences  (*p* values) |
| --- | --- | --- | --- | --- |
| Pre-supplementary motor area | L/R | 46190 (4664) | 43968 (3886) | 0.054 |
| Inferior frontal gyrus | R | 5032 (682) | 5082(634) | 0.774 |
| Caudate nucleus | R | 3324 (395) | 3593 (580) | 0.044 |

Group differences, *p* values of two-sample *t* tests; asterisks, *p*<0.017 (Bonferroni correction for three tests).

**SFig.5:** Correlation between the tissue volume and stopping-related activity of the right caudate nucleus is driven by an outlier (red circle).


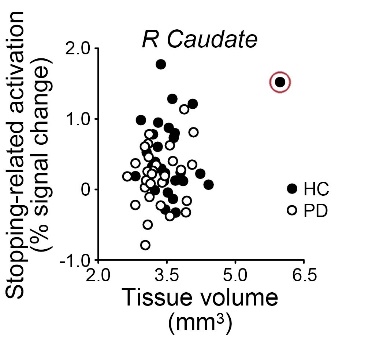


1. **No contribution of tissue volume to inhibitory functions of the fronto-subthalamic pathways**

We examined whether the frontal tissue volume contributed to inhibitory functions of the fronto-subthalamic pathways. In HCs, we asked (a) whether SSRT correlated with the right IFG tissue volume, in addition to the stopping-related activity of the right IFG and right STN (stepwise regression, *p*<0.05); and (b) whether the stopping-related activity correlated with the right IFG tissue volume, in addition to the LC CNR (stepwise regression, *p*<0.05). The brain-behavior regression model (*F*(2,26)=6.13, *p*=0.007, *R*^2^=0.32) included the stopping-related activity of the right IFG and right STN but not the right IFG tissue volume (*p*=0.579). The structure-function regression model (*F*(1,27)=4.76, *p*=0.038, *R*^2^=0.15) included the LC CNR but not the right IFG tissue volume (*p*=0.491).

In PD patients, we asked (a) whether SSRT correlated with the preSMA tissue volume, in addition to the preSMA-right STN functional connectivity (stepwise regression, *p*<0.05); and (b) whether the preSMA-right STN functional connectivity correlated with the preSMA tissue volume (Pearson correlation, *p*<0.05). The brain-behavior regression model (*F*(1,27)=4.44, *p*=0.045, *R*^2^=0.14) included the preSMA-right STN functional connectivity but not the preSMA tissue volume (*p*=0.375). The structure-function correlation was not significant (*p*=0.585).

Therefore, we found no contribution of tissue volume on inhibitory functions of the fronto-subthalamic pathways.

1. **Effects of the lateralization of motor symptoms on stop-signal inhibition**

Although stop-signal inhibition relies on a right-lateralized brain network,(Jahfari et al., 2011) previous studies reported similar stop-signal task performance in PD patients with left-dominant motor symptoms (PD-LD) and those with right-dominant motor symptoms (PD-RD).(Mirabella, Fragola, Giannini, Modugno, & Lakens, 2017) As a validity check, we examined differences between PD-LD and PD-RD patients in terms of task performance, stopping-related activity, and functional connectivity in the fronto-subthalamic pathways.

Scores of each body side were summed up from items 3-8 and 15-17 of the UPDRS Part III subscale. The side with a higher motor score was defined as the most affected side. In PD patients, 12/29 patients showed left-side dominance, and 16/29 patients showed right-sided dominance. One patient with no lateralization was excluded from this analysis. STable 2 shows the demographic, clinical, and neuropsychological data of the two subgroups.

We explored group differences in task performance, stopping-related activity, and functional connectivity using two-sample *t* tests (*p*<0.05). PD-RD patients tended to show longer SSRT (STable 3) and lower stopping-related activity than PD-LD patients (STable 4). This observation is against the assumption that PD-LD patients suffer from more severe deficits in the right-lateralized brain network for response inhibition.

**STable 2:** Demographic, clinical, neuropsychological, and neuromelanin data (means, SDs, and subgroup differences)

| Features/Measures | PD-LD  (*n*=12) | | PD-RD  (*n*=16) | Subgroup differences  (*p* values) |
| --- | --- | --- | --- | --- |
| Male/Female | 8/4 | | 8/8 | 0.378 |
| Age (years) | 61.8 (5.8) | | 66.8 (6.6) | 0.048 |
| Education (years) | 9.8 (2.0) | | 10.9 (2.7) | 0.255 |
| Body Mass Index | 24.3 (3.5) | | 23.2 (3.7) | 0.438 |
| *Motor symptoms* | |  | |  |
| Age of onset (years) | 59.7 (4.4) | | 65.3 (6.3) | 0.015 |
| Disease duration (years) | 3.4 (2.1) | | 1.7 (2.1) | 0.345 |
| Hoehn and Yahr stage | 1.9 (0.7) | | 1.7 (0.6) | 0.350 |
| UPDRS III score | 26.7 (11.1) | | 22.5 (12.3) | 0.363 |
| *Levodopa equivalent daily dose* | | | |  |
| Total (mg/day) | 353.1 (172.0) | | 262.5 (194.6) | 0.212 |
| Levodopa (mg/day) | 208.3 (147.5) | | 153.1 (142.9) | 0.327 |
| D2/3 receptor agonists (mg/day) | 78.1 (47.4) | | 56.3 (47.4) | 0.238 |
| *Non-motor functions* | |  | |  |
| Geriatric Depression Scale score | 5.7 (3.0) | | 4.9 (2.7) | 0.475 |
| REM Sleep Behavior Disorder Screening Questionnaire score | 5.6 (3.8) | | 5.4 (3.5) | 0.881 |
| Epworth Sleep Scale score | 8.3 (6.3) | | 4.1 (2.7) | 0.026 |
| Montreal Cognitive Assessment Basic score | 26.5 (1.8) | | 25.4 (1.9) | 0.119 |
| *Neuromelanin signals* | | |  |  |
| SN area (mm^2^) | 38.1 (21.4) | | 26.3 (10.4) | 0.101 |
| LC contrast-to-noise ratio | 3.1 (1.1) | | 3.3 (1.2) | 0.657 |

Group differences, *p* values of two-sample *t* or Chi-square tests as appropriate; asterisks, *p*<0.003 (Bonferroni correction for 17 tests); UPDRS, Unified Parkinson’s Disease Rating Scale.

**STable 3:** Stop-signal task performance (means, SEMs, and subgroup differences)

| Parameters | PD-LD  (*n*=12) | PD-RD  (*n*=16) | Subgroup differences  (*p* values) |
| --- | --- | --- | --- |
| Rate of Go omissions (%) | 1.4 (0.4) | 3.8 (1.6) | 0.225 |
| Rate of Go commission errors (%) | 3.4 (1.2) | 2.9 (1.0) | 0.753 |
| Reaction time of correct Go (ms) | 579 (19) | 596 (26) | 0.611 |
| P(respond \| stop-signal) | 0.46 (0.02) | 0.48 (0.02) | 0.303 |
| Stop-signal delay (ms) | 285 (19) | 250 (24) | 0.290 |
| Reaction time of failed Stop (ms) | 518 (16) | 547 (11) | 0.387 |
| SSRT (ms) | 277 (9) | 344 (24) | 0.027 |

Group differences, *p* values of two-sample *t* tests.

**STable 4:** Stopping-related activity and functional connectivity in the fronto-subthalamic pathways (means, SEMs, and subgroup differences)

| Parameters | PD-LD  (*n*=12) | PD-RD  (*n*=16) | Subgroup differences  (*p* values) |
| --- | --- | --- | --- |
| *Stopping-related percent signal change (%)* | | |  |
| preSMA | 0.41 (0.29) | 0.33 (0.32) | 0.494 |
| R IFG | 0.94 (0.28) | 0.67 (0.37) | 0.043 |
| R STN | 0.28 (0.81) | 0.10 (0.88) | 0.566 |
| *Functional connectivity (Fisher’s z)* | |  |  |
| preSMA - R STN | 0.12 (0.10) | 0.11 (0.09) | 0.679 |
| R IFG - R STN | 0.07 (0.08) | 0.06 (0.11) | 0.925 |

Group differences, *p* values of two-sample *t* tests.

1. **Additional correlation analyses**

**STable 5:** No correlation between the stopping-related right IFG activity and preSMA-right STN functional connectivity (*r* and *p* values)

| Groups | *r* values | *p* values |
| --- | --- | --- |
| HCs | 0.18 | 0.359 |
| PD | -0.27 | 0.890 |

**STable 6:** No correlation between the SN area and preSMA-STN pathway (*p* values)

| SN area x | PD  (*n*=29) | HC  (*n*=29) |
| --- | --- | --- |
| preSMA activity | 0.112 | 0.942 |
| R STN activity | 0.225 | 0.211 |
| preSMA - R STN connectivity | 0.816 | 0.787 |

**STable 7:** No correlation between dopaminergic drugs and fronto-caudate pathways (*p* values)

| Parameters | Levodopa  (*n*=29) | D2/3 receptor agonists  (*n*=29) |
| --- | --- | --- |
| R caudate activity | 0.270 | 0.063 |
| preSMA - R caudate connectivity | 0.740 | 0.138 |
| R IFG – R caudate connectivity | 0.485 | 0.044 |

1. **References**

Destrieux, C., Fischl, B., Dale, A., & Halgren, E. (2010). Automatic parcellation of human cortical gyri and sulci using standard anatomical nomenclature. *Neuroimage, 53*(1), 1-15.

Fischl, B. (2012). FreeSurfer. *Neuroimage, 62*(2), 774-781.

Jahfari, S., Waldorp, L., van den Wildenberg, W. P. M., Scholte, H. S., Ridderinkhof, K. R., & Forstmann, B. U. (2011). Effective connectivity reveals important roles for both the hyperdirect (fronto-subthalamic) and the indirect (fronto-striatal-pallidal) fronto-basal ganglia pathways during response inhibition. *Journal of Neuroscience, 31*(18), 6891-6899.

Li, Y., Wang, C., Wang, J., Zhou, Y., Ye, F., Zhang, Y., . . . Jin, L. (2019). Mild Cognitive Impairment in De Novo Parkinson’s Disease: A Neuromelanin MRI Study in Locus Coeruleus. *Movement Disorders, 34*(6), 884-892.

Minkova, L., Habich, A., Peter, J., Kaller, C. P., Eickhoff, S. B., & Klöppel, S. (2017). Gray matter asymmetries in aging and neurodegeneration: A review and meta-analysis. *Human Brain Mapping, 38*(12), 5890-5904.

Mirabella, G., Fragola, M., Giannini, G., Modugno, N., & Lakens, D. (2017). Inhibitory control is not lateralized in Parkinson's patients. *Neuropsychologia, 102*, 177-189.

Ye, R., Rua, C., O'Callaghan, C., Jones, P. S., Hezemans, F. H., Kaalund, S. S., . . . Rowe, J. B. (2021). An in vivo probabilistic atlas of the human locus coeruleus at ultra-high field. *Neuroimage, Epub*. doi:doi: 10.1016/j.neuroimage.2020.117487
